# Sinus Tarsi Versus Extensile Lateral Approach for Sanders Type II–IV Calcaneal Fractures: A Comparative Analysis of Functional Outcomes, Return to Work, and Cosmetic Parameters

**DOI:** 10.3390/jcm15093420

**Published:** 2026-04-29

**Authors:** Sahan Guven, Izzet Bingol, Cem Demir, Umut Oktem, Yasin Erdogan, Ali Said Nazligul

**Affiliations:** 1 Department of Orthopedics and Traumatology, Ankara Bilkent City Hospital, 06800 Ankara, Turkey; bingolizzet@gmail.com (I.B.); cemdemir0202@gmail.com (C.D.); umutoktem91@gmail.com (U.O.); 2 Department of Orthopedics and Traumatology, Faculty of Medicine, Ankara Yıldırım Beyazıt University, 06800 Ankara, Turkey; 3 Department of Orthopedics and Traumatology, Etlik City Hospital, 06170 Ankara, Turkey; yasin-erdgn@hotmail.com; 4 Department of Orthopedics and Traumatology, Sincan Training and Research Hospital, 06949 Ankara, Turkey; alisaid012@hotmail.com

**Keywords:** calcaneal fracture, sinus tarsi approach, extensile lateral approach, functional outcomes, return to work, shoe size change

## Abstract

**Background/Objectives:** The optimal surgical approach for displaced intra-articular calcaneal fractures (DIACFs) remains controversial. While the extensile lateral approach (ELA) has traditionally been preferred for complex fractures, the sinus tarsi approach (STA) has gained popularity due to its potentially lower soft tissue morbidity. However, comparative data focusing on patient-centered outcomes remain limited. This study aimed to compare clinical, radiological, functional, cosmetic, and complication outcomes between STA and ELA in Sanders type II–IV DIACFs. **Methods**: A retrospective comparative cohort study was conducted including patients treated with open reduction and internal fixation using either STA or ELA between February 2019 and October 2024. Functional outcomes were assessed using the AOFAS Ankle–Hindfoot Score and VAS. Radiological evaluation included Böhler and Gissane angles measured preoperatively, early postoperatively, and at final follow-up. Patient-centered outcomes included time to full weight bearing, return to work, heel width difference, and changes in shoe size. Complications were recorded throughout follow-up. **Results**: Baseline demographic and fracture characteristics were comparable between groups. Patients treated with STA demonstrated significantly shorter hospital stay, earlier progression to full weight bearing, and earlier return to work (*p* < 0.001). Functional outcomes favored STA, with significantly lower VAS scores and higher AOFAS scores at final follow-up (*p* < 0.05). No significant differences were observed between groups regarding Böhler or Gissane angles at any time point (*p* > 0.05). Wound-related complications were significantly more frequent in the ELA group (*p* = 0.018), although overall complication rates were comparable. **Conclusions**: The sinus tarsi approach was associated with comparable radiological restoration to the extensile lateral approach while demonstrating earlier functional recovery and lower wound-related morbidity. Given the retrospective and non-randomized design, these findings should be interpreted as associations rather than causal effects. STA may represent a safe and effective surgical option in appropriately selected Sanders type II–IV intra-articular calcaneal fractures.

## 1. Introduction

Displaced intra-articular calcaneal fractures (DIACFs) represent one of the most functionally debilitating injuries among foot and ankle traumas [1]. Typically resulting from high-energy mechanisms, these fractures disrupt the congruity of the posterior facet, reduce calcaneal height, increase heel width, and lead to axial deformities, ultimately predisposing patients to chronic pain, gait impairment, and long-term work disability [2]. The computed tomography-based Sanders classification is widely used to characterize fracture severity and predict prognosis; notably, increasing articular comminution in Sanders type II–IV fractures has been associated with progressively worse functional outcomes [3,4].

The primary objective of surgical management in DIACFs is to achieve anatomical reduction of the posterior facet, restore calcaneal height and length, reduce heel width, and preserve subtalar joint biomechanics [5]. For many years, the extensile lateral approach (ELA) has been considered the gold standard due to the wide surgical exposure it provides. However, ELA has been associated with significant soft tissue complications, including wound edge necrosis, wound-healing problems, and deep infection [6]. In an effort to reduce these complication rates, the sinus tarsi approach (STA) was developed to allow direct visualization of the posterior facet through a more limited soft tissue dissection. Recent studies, particularly in Sanders type II–III fractures, have reported that STA offers comparable radiological and functional outcomes to ELA, while demonstrating lower rates of wound-related complications [7,8].

However, the majority of comparative studies evaluating the STA and the ELA have primarily focused on relatively less severe fracture patterns and have limited their assessments to functional scores and radiographic parameters [9]. In contrast, clinically relevant patient-centered outcomes such as time to return to work, time to full weight bearing, and length of hospital stay have been addressed in a limited number of studies and are often reported heterogeneously [10]. Similarly, heel widening and changes in shoe size, which represent characteristic sequelae of calcaneal fractures and may substantially affect patients’ daily functioning and cosmetic satisfaction, have largely been overlooked in approach-based comparative analyses [9,11]. Therefore, there remains a need for studies that comprehensively compare STA and ELA in Sanders type II–IV intra-articular calcaneal fractures, not only in terms of radiological and functional scores but also with respect to clinically relevant parameters such as return to work, time to full weight bearing, cosmetic outcomes, and complication profiles within a consistent methodological framework.

The primary hypothesis of this study was that, in Sanders type II–IV intra-articular calcaneal fractures, the sinus tarsi approach (STA) would provide at least comparable potentially superior radiological and functional outcomes with lower complication rates compared with the extensile lateral approach (ELA). In addition, it was hypothesized that STA would yield more favorable results in clinically and cosmetically relevant patient-centered parameters, including time to full weight bearing, time to return to work, heel widening, and changes in shoe size. Accordingly, the aim of this study was to comprehensively compare the clinical, radiological, functional, and cosmetic outcomes of patients with Sanders type II–IV DIACFs treated surgically using either STA or ELA within a consistent methodological framework.

## 2. Materials and Methods

This study was designed as a retrospective comparative cohort study evaluating patients who underwent surgical treatment for intra-articular calcaneal fractures at our institution between February 2019 and October 2024. Patients with Sanders type II, III, or IV intra-articular calcaneal fractures who were treated with open reduction and internal fixation (ORIF) using either the extensile lateral approach or the sinus tarsi approach were included in the study. All data were obtained from the institutional clinical database, including medical records, operative reports, radiological imaging archives, and outpatient follow-up documentation. The study protocol was approved by the Institutional Review Board (IRB) (Approval No: 2-25-1103, 16 April 2025). Given the retrospective design of the study, the requirement for additional informed consent was waived by the ethics committee. All procedures were conducted in accordance with the principles of the Declaration of Helsinki.

Inclusion criteria comprised patients aged 18 years or older who underwent surgical treatment using either the extensile lateral approach (ELA) or the sinus tarsi approach (STA) for computed tomography-confirmed Sanders type II–IV intra-articular calcaneal fractures. Eligible patients were required to have a minimum of 12 months of clinical and radiological follow-up and complete clinical and radiographic data available for postoperative evaluation. Exclusion criteria included Sanders type I or extra-articular calcaneal fractures, open fractures, bilateral calcaneal fractures, prior history of calcaneal, subtalar, or hindfoot surgery on the affected side, and concomitant injuries in the same extremity that could potentially influence clinical outcomes (e.g., ipsilateral talus fractures, tibial pilon fractures, or ankle fractures). Patients with insufficient follow-up duration or incomplete clinical and/or radiological data were also excluded from the study.

Demographic characteristics of the patients were recorded. Body mass index (BMI, kg/m^2^) was calculated for all cases. Comorbidities, particularly the presence of diabetes mellitus, were documented based on medical records. The mechanism of injury was categorized as fall from height, direct trauma, motor vehicle accident, or other causes. Smoking status was also recorded. Follow-up duration was defined as the interval between the date of surgery and the most recent clinical evaluation and was documented in months. These variables were considered potential confounding factors and were included in the comparative analyses between the two surgical approach groups.

All surgical procedures were performed by orthopedic surgeons experienced in foot and ankle surgery under spinal anesthesia. Patients were positioned in the lateral decubitus position on a radiolucent operating table, and a pneumatic tourniquet was applied at a constant pressure of 300 mmHg. Prophylactic antibiotic therapy consisting of 2 g of intravenous cefazolin was administered approximately 30 min prior to skin incision in all patients.

In patients treated with the extensile lateral approach (ELA), a standard L-shaped lateral incision was utilized. The skin and subcutaneous tissues were elevated as a full-thickness flap to allow wide exposure of the lateral wall, posterior facet, and subtalar joint surface. Care was taken to protect the peroneal tendons and the sural nerve during lateral wall exposure. The posterior facet fragments were anatomically reduced under direct visualization and temporarily stabilized with Kirschner wires. Restoration of calcaneal height, length, and alignment was subsequently confirmed before final fixation. Definitive fixation was achieved using an anatomical lateral calcaneal plate with an appropriate number of screws.

**Figure 1:**
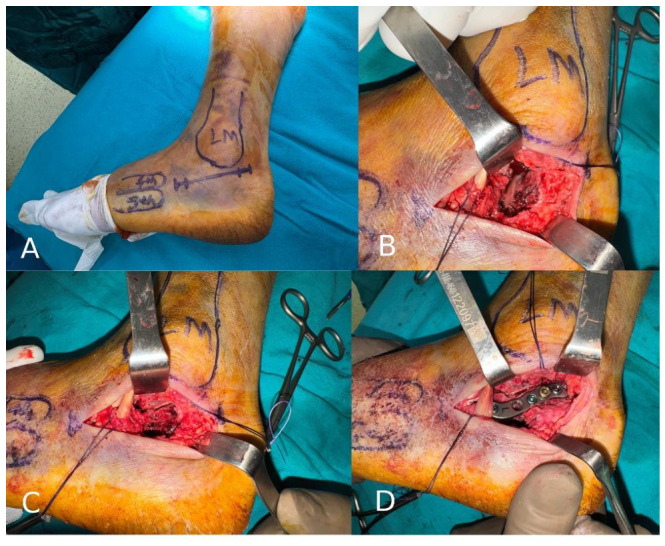
Intraoperative steps of the sinus tarsi approach: (**A**) skin marking, (**B**) fracture exposure, (**C**) fracture reduction, and (**D**) final fixation.

In patients treated with the sinus tarsi approach (STA), a limited oblique lateral incision was made extending from the distal aspect of the lateral malleolus toward the base of the fourth metatarsal through the sinus tarsi. The posterior facet was directly visualized via the sinus tarsi, allowing access to the subtalar joint surface with minimal soft tissue dissection. The posterior facet fragments were anatomically reduced under fluoroscopic guidance and temporarily stabilized, after which restoration of calcaneal height and alignment was assessed. Definitive fixation was achieved using screws supporting the posterior facet and/or low-profile plate systems, in addition to two 6.5 mm cannulated screws inserted laterally to restore calcaneal length and alignment. Lateral wall exposure was kept limited, and the peroneal tendons were preserved to maintain maximal soft tissue integrity. The key intraoperative steps of the sinus tarsi approach are demonstrated in Figure 1.

In both approaches, the quality of reduction and implant positioning were confirmed intraoperatively using fluoroscopy. The postoperative rehabilitation protocol was identical for both groups. Postoperatively, patients were mobilized without weight bearing for the first 8 weeks, during which passive and active range-of-motion exercises were initiated to preserve ankle and subtalar joint mobility. Progression to full weight bearing was planned gradually based on clinical assessment and radiographic evidence of fracture healing. The decision to allow progression to full weight bearing was made by the treating surgeon according to pain level, clinical stability, and radiographic signs of union.

The selection of surgical approach (sinus tarsi vs. extensile lateral) was not based on a standardized protocol but was primarily determined by the operating surgeon’s preference and experience. Each surgeon consistently performed the technique with which they were most familiar. Both surgical approaches were applied contemporaneously during the study period across a comparable distribution of fracture types.

### 2.1. Clinical and Functional Evaluations

Functional outcomes were assessed using the American Orthopaedic Foot and Ankle Society (AOFAS) Ankle–Hindfoot Score to evaluate foot and hindfoot function. The AOFAS score is a widely used functional assessment tool in ankle and hindfoot pathologies, consisting of three subdomains: pain (40 points), function (50 points), and alignment (10 points), with a total possible score of 100 points [12]. Pain intensity was quantified using the Visual Analog Scale (VAS), which measures patients’ perceived pain on a continuous scale ranging from 0 (no pain) to 10 (worst imaginable pain). The VAS is considered a reliable and valid method for pain assessment in orthopedic trauma studies [13]. Blinding of clinical outcome assessment was not feasible because the surgical incisions differed substantially between the two approaches and were readily identifiable during follow-up examinations.

With respect to clinical outcomes, time to return to work was recorded in months and defined as the interval between surgery and the patient’s first return to occupational activity. Time to full weight bearing was determined in weeks based on the time point at which full weight bearing was permitted by the treating surgeon according to clinical examination findings and radiographic evidence of fracture healing. As a cosmetic and functional patient-reported outcome, changes in shoe size were assessed by comparing the pre-injury shoe size with the shoe size reported at final follow-up. Heel width difference was evaluated during clinical follow-up examinations. Patients were asked to stand bearing full weight, and the contour of each heel was traced onto a sheet of paper. Transverse width was measured at the widest portion of the heel [14]. Measurements were compared with the contralateral, uninjured side, and heel width difference was recorded in millimeters.

Length of hospital stay was calculated in days, defined as the interval between the date of surgery and the date of discharge. Complications were defined as soft tissue or wound-related problems (superficial wound dehiscence, delayed wound healing, skin edge necrosis), subtalar arthritis, sural nerve injury, and implant-related symptoms, and were recorded throughout the follow-up period.

### 2.2. Radiological Evaluations

For radiological assessment, preoperative, early postoperative, and final follow-up images of all patients were analyzed. In the preoperative period, the Böhler angle and the Gissane angle were measured to evaluate calcaneal morphology and fracture-related deformity. The Böhler angle was defined as the angle formed on lateral foot radiographs by lines drawn between the posterior tuberosity, the highest point of the posterior facet, and the anterior process, reflecting calcaneal height [15]. The Gissane angle, formed between the posterior facet and the anterior process, was considered a critical angle indicative of disruption of the subtalar joint surface [16].

**Figure 2:**
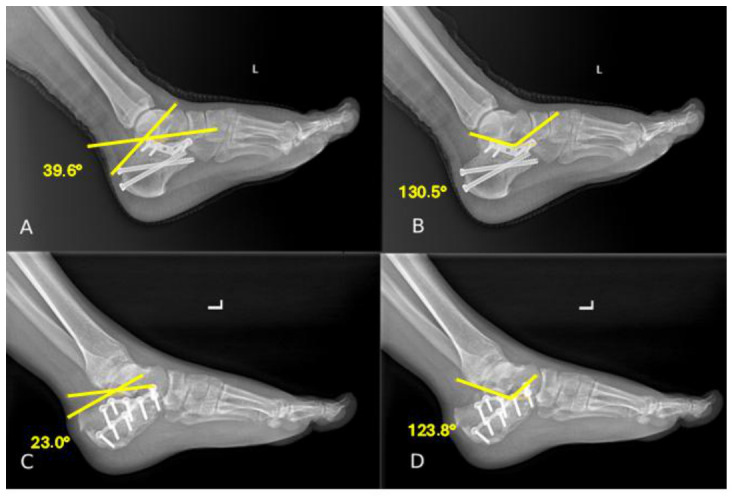
Postoperative lateral foot radiographs demonstrating Böhler and Gissane angle measurements. The yellow lines indicate the reference lines used for Böhler and Gissane angle measurements. (**A**) Postoperative Böhler angle measurement in a patient treated with the sinus tarsi approach. (**B**) Postoperative Gissane angle measurement in the same patient. (**C**) Postoperative Böhler angle measurement in a patient treated with the extensile lateral approach. (**D**) Postoperative Gissane angle measurement in the same patient.

To evaluate the success of surgical reduction, the Böhler and Gissane angles were remeasured on early postoperative radiographs obtained within the first week after surgery and compared with preoperative values (Figure 2). To assess maintenance of reduction, the same measurements were repeated on radiographs obtained at the final clinical follow-up.

All measurements were performed on standardized lateral foot radiographs in which the posterior tuberosity, posterior facet, and anterior process of the calcaneus were clearly visualized. The same anatomical reference points were used for each patient to ensure consistency. Böhler and Gissane angles measured in the preoperative, early postoperative, and final follow-up periods were comparatively analyzed between the two surgical approach groups.

To assess the reliability of radiological measurements, all Böhler and Gissane angles were independently measured by two experienced orthopedic trauma surgeons who were blinded to both the surgical approach and clinical outcomes. Interobserver reliability was evaluated using the Intraclass Correlation Coefficient (ICC) based on the measurements obtained by the two observers. For intraobserver reliability assessment, each observer repeated the measurements on radiographs from 30 randomly selected patients after a two-week interval, without access to prior measurements.

Interobserver reliability demonstrated good agreement, with an ICC of 0.87 for the Böhler angle and 0.85 for the Gissane angle. Intraobserver reliability was also high; ICC values for the Böhler angle were 0.90 and 0.88 for the two observers, respectively, while corresponding ICC values for the Gissane angle were 0.89 and 0.87.

### 2.3. Statistical Analysis

Statistical analyses were performed using IBM SPSS Statistics version 25 (IBM Corporation, Armonk, NY, USA). The normality of continuous variables was assessed using the Shapiro–Wilk test. Since most continuous variables did not show a normal distribution, data were presented as median and interquartile range (25th–75th percentiles). Categorical variables were expressed as frequency (*n)* and percentage (%). Intergroup comparisons between the sinus tarsi and lateral extensile groups were performed using the Mann–Whitney U test for continuous variables. Categorical variables were compared using the chi-square test or Fisher’s exact test, as appropriate, depending on expected cell counts. Radiological parameters evaluated at multiple time points, including Böhler and Gissane angles, were adjusted for multiple comparisons using the Bonferroni correction, with an adjusted significance level of *p* < 0.0167. Functional outcome measures (VAS and AOFAS scores) were predefined as primary outcomes and were therefore not adjusted for multiple comparisons. A *p* value <0.05 was considered statistically significant unless otherwise specified.

Due to the retrospective design of the study, no a priori sample size calculation was performed. However, a post hoc power analysis was conducted using G*Power version 3.1 (Heinrich Heine University, Düsseldorf, Germany) to evaluate the strength of the observed results. Two-tailed independent-samples comparisons (α = 0.05) were used for key outcome variables based on the observed effect sizes (Cohen’s d). The achieved powers were 100.0% for length of hospital stay (d = 1.90), 99.6% for time to full weight bearing (d = 0.99), 87.4% for return to work (d = 0.66), 87.3% for VAS (d = 0.66), and 95.1% for AOFAS (d = 0.77). In contrast, heel width difference showed a small effect size (d = 0.20) with low achieved power (15.3%).

Radiological measurements, including Böhler and Gissane angles, were independently performed by two experienced orthopedic surgeons who were blinded to the clinical outcomes and surgical approach. To assess interobserver reliability, measurements obtained by the two observers were compared. To assess intraobserver reliability, one observer repeated all radiological measurements after a minimum interval of two weeks, blinded to the initial measurements. Interobserver and intraobserver reliability for radiological measurements were evaluated using the intraclass correlation coefficient (ICC) with a two-way random-effects model and absolute agreement definition.

## 3. Results

Baseline demographic and fracture characteristics were comparable between the two groups. No statistically significant differences were observed regarding age, body mass index, side, smoking status, or Sanders classification (*p* > 0.05). However, patients treated with the sinus tarsi approach demonstrated significantly shorter hospital stay, earlier full weight bearing, and earlier return to work compared with those treated with the lateral extensile approach (*p* < 0.001) (Table 1).

Functional outcomes favored the sinus tarsi group, with significantly lower VAS scores and higher AOFAS scores at final follow-up (*p* < 0.05). Intergroup comparison revealed no significant differences in Böhler or Gissane angles at any time point (*p* > 0.05 for all) (Table 2).

The overall complication rate was lower in the sinus tarsi group compared with the lateral extensile group; however, this difference did not reach statistical significance (12.5% vs. 25.6%, *p* = 0.118). Wound-related complications were significantly more frequent in the lateral extensile group (20.9% vs. 4.2%, *p* = 0.018). Regarding heel width difference, in the sinus tarsi group, the difference was 0 mm in 45.8% of patients, 0.5 mm in 22.9%, 1.0 mm in 22.9%, 1.5 mm in 4.2%, and 2.0 mm in 4.2%. In the lateral extensile group, heel width difference was 0 mm in 44.2% of patients, 0.5 mm in 27.9%, 1.0 mm in 23.3%, 1.5 mm in 2.3%, and 2.0 mm in 2.3%. No significant differences were observed between the groups regarding infection, sural nerve injury, shoe size change or heel width difference (*p* > 0.05 for all) (Table 3).

## 4. Discussion

In the surgical management of intra-articular calcaneal fractures, the sinus tarsi approach (STA) has generally been recommended for less severe fracture patterns, whereas the extensile lateral approach (ELA) is traditionally preferred for more complex fractures [9,17,18]. The findings of the present study suggest that this conventional paradigm may warrant reconsideration. Patients treated with STA demonstrated earlier progression to full weight bearing and return to work, lower postoperative pain levels, and superior functional scores, while no significant differences were observed between the two approaches in terms of radiological parameters. Furthermore, the higher incidence of wound-related complications in the ELA group supports the potential biological advantages of the minimally invasive approach.

Recent comparative studies and meta-analyses have demonstrated that the sinus tarsi approach provides at least comparable, and in some reports potentially superior, functional outcomes compared with the extensile lateral approach [19,20]. In particular, evaluations based on AOFAS and VAS scores have indicated that STA, performed with more limited soft tissue dissection, may offer improved early pain control and functional recovery [9,21]. Consistent with these findings, patients in the STA group in the present study demonstrated lower VAS scores and higher AOFAS scores, aligning with the trends reported in the existing literature.

However, most studies in the literature have primarily evaluated functional outcomes using score-based assessments, whereas patient-centered clinical parameters such as time to full weight bearing, length of hospital stay, and time to return to work have been reported less consistently [2,22,23]. These variables more directly reflect the impact of surgical treatment on daily functioning and socioeconomic recovery. In the present study, patients treated with STA demonstrated shorter hospital stays, earlier progression to full weight bearing, and earlier return to work, suggesting that the choice of surgical approach may influence the practical trajectory of clinical recovery.

With regard to radiological outcomes, current evidence suggests that the sinus tarsi approach (STA) can achieve anatomical restoration comparable to that of the extensile lateral approach (ELA) [9]. In the meta-analysis by Yao et al., no significant difference was reported between the two approaches in terms of Böhler angle restoration [20]; similarly, more comprehensive analyses have demonstrated no statistically significant differences in either Böhler or Gissane angles [24]. In our study, no significant differences were observed between the groups in preoperative, early postoperative, or final follow-up radiological parameters. These findings indicate that while STA may confer early clinical advantages, it does not compromise the quality of radiological reduction.

With respect to complications, several studies have reported that the sinus tarsi approach is associated with lower rates of wound-healing problems compared with the extensile lateral approach [25,26,27]. Meta-analyses have suggested that STA may reduce postoperative wound complications [20], and similar trends have been observed in retrospective comparative studies [6,19]. Although no significant difference was identified in overall complication rates in our study, wound-related problems were more frequently observed in the ELA group, consistent with the existing literature. These findings underscore the importance of carefully considering soft tissue morbidity when selecting the surgical approach.

One of the strengths of this study is the evaluation of patient-centered parameters that have been relatively underreported in the literature, such as time to full weight bearing, time to return to work, and changes in shoe size. However, the retrospective design, single-center data, and limited sample size may restrict the generalizability of the findings. Another important limitation of this study is the potential for selection bias due to the retrospective design and non-randomized allocation of surgical approach. The choice of technique was surgeon-dependent, which may have influenced certain clinical outcomes. Although baseline fracture characteristics were comparable between groups, this potential bias should be considered when interpreting the results. In addition, multivariate analysis was not performed because of the limited sample size and the low number of outcome events, particularly for complications, which may have increased the risk of overfitting and unstable estimates. Therefore, the potential influence of residual confounding factors cannot be excluded.

Despite its widespread use in foot and ankle studies, the AOFAS score has been criticized for limited measurement precision, inclusion of physician-reported components, low construct validity when correlated with validated health-related quality-of-life measures, and suboptimal internal consistency and test–retest reliability compared with modern patient-reported outcome measures [28,29,30]. Therefore, the AOFAS findings in the present study should be interpreted with some caution, and future studies may benefit from incorporating additional validated patient-reported outcome instruments.

Furthermore, subgroup analyses according to Sanders classification could not be performed because the number of patients in each subgroup, particularly Sanders type IV fractures, was too small to allow reliable statistical comparisons. Therefore, the applicability of the sinus tarsi approach in more complex fracture patterns should be interpreted with caution. Another limitation of this study is that detailed information regarding occupational demands and the nature of return to work could not be consistently obtained because of the retrospective design. Therefore, return to work was defined as the first return to any occupational activity, regardless of whether this represented full-duty or modified work. In addition, postoperative CT scans were not routinely obtained because of concerns regarding additional radiation exposure and cost. Therefore, postoperative articular congruity, residual posterior facet step-off, and reduction quality could not be evaluated in a standardized manner for all patients. Instead, reduction quality was assessed using routinely obtained radiographic parameters, including Böhler and Gissane angles. Moreover, the post hoc power analysis demonstrated that the study was underpowered to detect differences in heel width difference, with an achieved power of 15.3%. Therefore, the absence of a statistically significant difference in cosmetic outcomes, including heel width and shoe size change, should be interpreted with caution. Finally, the absence of long-term outcomes, particularly regarding subtalar joint degeneration, represents another important limitation. Therefore, prospective, multicenter studies with larger sample sizes are warranted to more clearly define the impact of surgical approach selection on clinical and functional outcomes.

## 5. Conclusions

This study suggests that the sinus tarsi approach is associated with earlier progression to full weight bearing and earlier return to work compared with the extensile lateral approach. However, given the retrospective and non-randomized design, these findings should be interpreted as associations rather than causal relationships. While STA provided at least comparable functional outcomes, no significant differences were observed between the two approaches in terms of radiological parameters. The higher incidence of wound-related complications in the ELA group suggests a potential soft tissue advantage of the minimally invasive approach. These findings support the consideration of STA as a safe and effective surgical option. While functional outcomes favored STA, conclusions regarding cosmetic outcomes remain limited due to insufficient statistical power.

## Figures and Tables

**Table 1:** Demographic and clinical characteristics of the study groups.

	Sinus Tarsi (*n* = 48)	Lateral Extensile *(n* = 43)	*p* Value
Age (years)	47.0 (42.0–58.0)	41.0 (40.0–58.5)	0.573 ^a^
BMI (kg/m^2^)	28.0 (21.0–30.0)	26.0 (24.0–29.5)	0.968 ^a^
Side (Right/Left)	24/23	20/23	0.826 ^b^
Smoking (No/Yes)	18/29	17/26	1.000 ^b^
Sanders classification (II/III/IV)	21/20/6	19/17/7	0.886 ^b^
Length of hospital stay (days)	6.0 (5.0–7.0)	9.0 (7.5–10.0)	<0.001 ^a^
Time to full weight bearing (weeks)	12.0 (10.0–14.5)	14.0 (12.0–15.0)	<0.001 ^a^
Return to work (months)	5.0 (4.0–6.0)	6.0 (5.0–7.5)	<0.001 ^a^

Values are presented as median (interquartile range) or number of patients. ^a^ Mann–Whitney U test, ^b^ Chi-square (χ^2^) test. BMI: Body mass index.

**Table 2:** Functional and radiological outcomes of the study groups.

	Sinus Tarsi (*n* = 48)	Lateral Extensile (*n* = 43)	*p* Value
VAS	2.0 (1.0–2.0)	3.0 (1.5–3.0)	0.004 ^a^
AOFAS	80.0 (78.0–88.0)	75.0 (70.0–83.0)	<0.001 ^a^
**Böhler angle**			
Preoperative	12.0 (3.0–15.5)	8.0 (3.0–15.0)	0.442 ^a†^
Postoperative	25.0 (21.0–32.0)	24.0 (21.0–27.0)	0.059 ^a†^
Final follow-up	23.0 (20.5–30.0)	22.0 (17.0–28.0)	0.060 ^a†^
**Gissane angle**			
Preoperative	142.0 (137.0–152.0)	139.0 (137.5–147.0)	0.186 ^a†^
Postoperative	132.0 (121.5–132.3)	129.0 (119.5–133.0)	0.529 ^a†^
Final follow-up	129.0 (119.8–132.0)	129.0 (122.0–134.0)	0.501 ^a†^

Values are presented as median (interquartile range) or number (%). ^a^ Mann–Whitney U test, ^†^ Bonferroni correction was applied for multiple comparisons of radiological parameters (adjusted significance level *p* < 0.0167). VAS: Visual Analog Scale. AOFAS: American Orthopaedic Foot and Ankle Society score.

**Table 3:** Complications and categorical outcomes of the study groups.

	Sinus Tarsi (*n* = 48)	Lateral Extensile (*n* = 43)	*p* Value
Any complication, *n* (%)	6 (12.5%)	11 (25.6%)	0.118 ^a^
Wound problem, *n* (%)	2 (4.2%)	9 (20.9%)	0.018 ^b^
Infection, *n* (%)	1 (2.1%)	3 (7.0%)	0.336 ^a^
Sural nerve injury, *n* (%)	1 (2.1%)	2 (4.7%)	0.598 ^a^
Shoe size change, *n* (%)	21 (43.8%)	24 (55.8%)	0.236 ^a^
Heel width difference (>0 mm), *n* (%)	22 (45.8%)	26 (60.5%)	0.236 ^a^

Values are presented as number (%). ^a^ Chi-square (χ^2^) test, ^b^ Fisher’s exact test.

## Data Availability

The data presented in this study are available upon request from the corresponding author.

## Abbreviations

The following abbreviations are used in this manuscript:

DIACF	displaced intra-articular calcaneal fracture
ELA	extensile lateral approach
STA	Sinus tarsi approach
AOFAS	American Orthopaedic Foot and Ankle Society
VAS	Visual analog scale
ORIF	open reduction and internal fixation
